# Effect of Flowering Shading on Grain Yield and Quality of Durum Wheat in a Mediterranean Environment

**DOI:** 10.3390/plants14010076

**Published:** 2024-12-29

**Authors:** Giancarlo Pagnani, Alfredo Lorenzo, Nausicaa Occhipinti, Lisa Antonucci, Sara D’Egidio, Fabio Stagnari, Michele Pisante

**Affiliations:** Department of Bioscience and Technologies for Food, Agriculture and Environment, University of Teramo, Via Balzarini, 1, 64100 Teramo, Italy; alorenzo@unite.it (A.L.); nocchipinti@unite.it (N.O.); lantonucci@unite.it (L.A.); sdegidio@unite.it (S.D.); mpisante@unite.it (M.P.)

**Keywords:** winter wheat, yield component analysis, wheat quality, nitrogen application, light-use efficiency

## Abstract

The phenomenon known as “dimming” or shading, caused by the increase in aerosols, air pollutants, and population density, is reducing global radiation, including both direct solar radiation and radiation scattered by the atmosphere. This phenomenon poses a significant challenge for agricultural production in many regions worldwide, with a global radiation decrease estimated between 1.4% and 2.7% per decade in areas between 25° N and 45° N. In particular, in Mediterranean regions, the production of durum wheat (*Triticum turgidum* L. subsp. *Durum*) is increasingly constrained by abiotic factors, such as spring/summer heat stress and drought, as well as reductions in solar radiation. Field experiments were conducted in Mosciano Sant’Angelo, Italy, over two cropping seasons (2016–2017 and 2017–2018) to evaluate the effects of photosynthetically active radiation (PAR) availability and nitrogen (N) fertilization on durum wheat. A split-plot design was used with two PAR levels (100% and 20% PAR) and three N rates (0, 100, and 250 kg ha^−1^). Results highlighted that full sunlight (NoSh) significantly increased grain yield (+25%), thousand kernel weight (+46%), and total gluten fractions (+16%) compared to shaded conditions (Sh). Chlorophyll content and NDVI values were highest under Sh combined with 250 kg N ha^−1^. Rainfall patterns strongly influenced productivity, with better vegetative growth in 2016–2017 and improved grain filling in 2017–2018. Nitrogen application significantly enhanced grain protein content, particularly under arid conditions. These findings emphasize the interaction between light availability and nitrogen management, suggesting that optimizing these factors can improve yield and quality in durum wheat under Mediterranean conditions.

## 1. Introduction

The increase in aerosols, air pollutants, and population density has led to a phenomenon known as dimming or shading, which refers to the reduction in global radiation, including both direct solar radiation and radiation scattered by the atmosphere. Aerosols and other particles from pollution, dust, or volcanic eruptions usually absorb solar energy and reflect sunlight back into space. These pollutants can also act as a nucleus for cloud formation as water droplets cluster around them. Consequently, an increased concentration of particles in the atmosphere leads to clouds consisting of a larger number of smaller droplets that reflect sunlight better. This results in more sunlight being reflected back into space and less reaching the Earth’s surface [1]. This phenomenon poses a significant challenge for crop production in many regions worldwide [2]. Research has shown that in areas between 25° N and 45° N, global radiation has decreased by about 1.4–2.7% per decade [3]. In particular, the lower Yangtze River Plain, which accounts for 15% of the total wheat-growing area in China, is a heavily industrialized zone with a high risk of air pollution. In this region, the dimming phenomenon is especially pronounced, with reductions in radiation exceeding 6% per decade [4,5]. Durum wheat yields and quality in the Mediterranean region are increasingly challenged by various abiotic factors, particularly spring and summer heat stress and drought. In addition, PAR in Mediterranean ecosystems is particularly important due to the unique climatic conditions in this region. These ecosystems are often characterized by high solar radiation and seasonal water scarcity. Understanding PAR in this context could shed light on how plants optimize their photosynthetic performance under stress conditions and adapt to fluctuations in light and water availability [6]. The International Food Policy Research Institute (IFPRI) predicts that these challenges are likely to intensify in the near future [7]. In addition, current climate models indicate that climate change is likely to have a negative impact on wheat phenology and yields [8,9]. Grain yields of durum wheat under optimal conditions are influenced by variations in solar radiation [10]. Variations in grain yield between different environments can often be attributed to differences in the number of grains per square meter [11]. Fischer [12] observed a linear relationship between the number of grains per unit area and the solar radiation received by the crop before anthesis. Reductions in ear dry weight and the number of fertile flowers at anthesis, as reported by Stockman et al. [13], explain the impact of shading on the number of grains per square meter. The most sensitive period for this effect occurs in the 20 days leading up to anthesis [14], during which the stem and ear elongate rapidly, and the florets are differentiating and maturing. During this phase, some of the flowers and shoots may die. This period is therefore crucial for determining the number of grains and the yield [15]. However, the grain-setting period should not be overlooked, as several authors have shown that post-anthesis stress also affects the number of grains per square meter [16,17]. The effects of other environmental factors on tiller production and survival have also been documented, including nutrient concentrations [18], carbon dioxide levels [19], radiation intensity [20], temperature [20], and light quality [21]. Several authors have analyzed the contributions of different shoot categories to grain yield in both common wheat [3,11,22,23,24,25] and durum wheat [10,26] but changes in the growth, gluten content, starch, and yield of different shoot categories under stress do not appear to have been reported. The objective was to evaluate the influence of variation in light intensity and nitrogen availability through (i) the quantification of the effect of photosynthetically active radiation (PAR) reduction on durum wheat yield, gluten content, and starch; (ii) analyzing the contribution of nitrogen fertilization, under reduced light and full sunlight conditions; (iii) assessing the interaction between reduced PAR and nitrogen fertilization on grain quality; and (iv) determining the effects of seasonal variations in rainfall and temperature on the light–nitrogen interaction. Within these objectives, the research aimed to contribute to optimizing durum wheat production in Mediterranean regions, particularly in the face of changing climatic conditions and potential reduction in solar radiation.

## 2. Results

### 2.1. Meteorological Data Analysis

**Figure 1:**
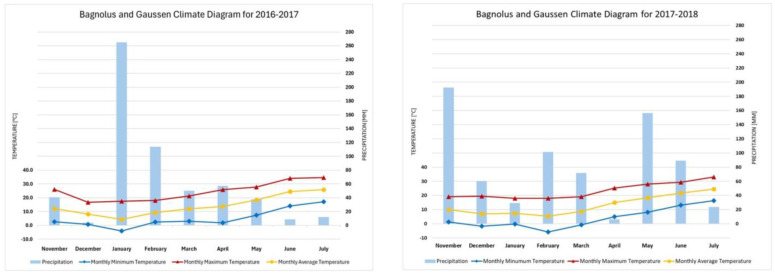
Monthly rainfall (represented by bars) and average monthly temperatures (indicated by dots) were recorded throughout the entire durum wheat crop cycle during the 2016–2017 and 2017–2018 growing seasons in the experimental area.

In the 2016–2017 season (Figure 1), the vegetative phase from emergence (mid-January) to heading (end of April) received significantly more cumulative rainfall (486.4 mm) than in 2017–2018 (208.4 mm), particularly in January (265 mm) and February (114 mm). This abundant precipitation likely provided favorable soil moisture for emergence and tillering, promoting strong vegetative growth and resulting in a higher number of ears per square meter, higher hectoliter weight, and greater grain protein content in 2017. Conversely, during the reproductive phase (heading to maturation, May and June), 2017–2018 experienced substantially more rainfall (245.5 mm) compared to 2016–2017 (46.4 mm), with higher precipitation in May (156.2 mm) and June (89.3 mm). This increased moisture could have enhanced flowering and grain filling, leading to longer ears in 2018. June presented arid conditions during the 2016–2017 season, with only 8.6 mm of precipitation. The lack of moisture during grain filling may have limited nutrient translocation to the grains, affecting yield and quality and contributing to shorter ear length despite higher ear density. April 2018 was also an arid month, possibly stressing the plants during late tillering and early heading, which could impact spikelet formation and result in fewer ears per square meter in 2018. Average monthly temperatures were similar between the two seasons during the vegetative and reproductive phases. However, the differing precipitation patterns suggest that 2016–2017 had more favorable moisture conditions during vegetative growth, enhancing plant establishment and ear density. Meanwhile 2017–2018 benefited from better moisture during reproductive stages, improving grain filling and ear length. The higher rainfall during grain filling in 2017–2018 may have diluted grain protein content and reduced hectoliter weight due to increased carbohydrate accumulation, whereas the arid conditions during grain filling in 2016–2017 could have concentrated proteins and increased grain density, resulting in higher protein content and hectoliter weight.

### 2.2. Chlorophylls and Carotenoids Content

The contents of ChlA, ChlB, ChlTot (ChlA + ChlB), and carotenoids in flag leaves are shown in Table 1 and Figure S1. For both years, chlorophyll a, b, and total concentrations saw statistically different increases as nitrogen levels increased (0, 100, and 250 kg ha^−1^) in both Sh and NoSh treatments, with the highest concentrations at 250_N. Specifically, in Chla, Sh was statistically significant compared to NoSh only in 2017 (+67%). Nitrogen provided the highest values in 250_N in 2017 and 100_N in 2018 (6.98 mg g^−1^ FW and 5.29 mg g^−1^ FW, respectively). The same trend was present in Chlb, except for PAR availability, where in both years, Sh was statistically different from NoSh (*p* < 0.01). Consequently, the highest values of Chl Tot were observed for Sh+250_N in 2017 and Sh+100_N in 2018 (*p* < 0.01). The interaction between PAR and N, in Chl Tot, was significant for both years, where we find the highest and lowest values in the Sh+250_N and NoSh+0_N conditions (11.05 mg g^−1^ FW and 2.43 mg g^−1^ FW, respectively). The NoSh condition was statistically significant for carotenoids compared with Sh in both years (+45% in 2017 and +44% in 2018), while 250_N was significant in 2017 compared with conditions 100_N and 0_N in 2017 (*p* < 0.01) and against 0_N in 2018 (*p* < 0.05). The interaction between PAR and N was not significant in 2017, in contrast to 2018, where we found the highest and lowest values in NoSh+250_N and Sh+0_N (0.54 mg g^−1^ FW and 0.28 mg g^−1^ FW, respectively).

### 2.3. Physiological Data

NDVI and SPAD assessments showed a trend in line with chlorophylls and carotenoids. Sh showed consistently higher high values than NoSh (Table 2). SPAD values were statistically significant compared to NoSh in both years (+13% in 2017 and +13% in 2018). Nitrogen availability gave increasing values as nitrogen levels increased (0, 100, and 250 kg/ha). The 250_N dose was statistically significant compared to 0_N in both years (*p* < 0.01). The interaction between PAR and N was significant for both years, with the highest and lowest values in the Sh+250_N condition (49.5 in 2017 and 48.7 in 2018, respectively). A similar trend was also recorded for NDVI values. The Sh condition was statistically different from NoSh only in 2017 (*p* < 0.01). The 250_N dose was statistically significant compared with 0_N in both years (*p* < 0.01). The interaction between PAR and N was statistically significant for both years, with the highest and lowest values in the Sh+250_N condition (0.884 in 2017 and 0.865 in 2018, respectively), and lowest in the NoSh+0_N condition (0.789 in 2017 and 0.790 in 2018, respectively).

### 2.4. Grain Yield and Thousand Kernel Weight 

**Figure 2:**
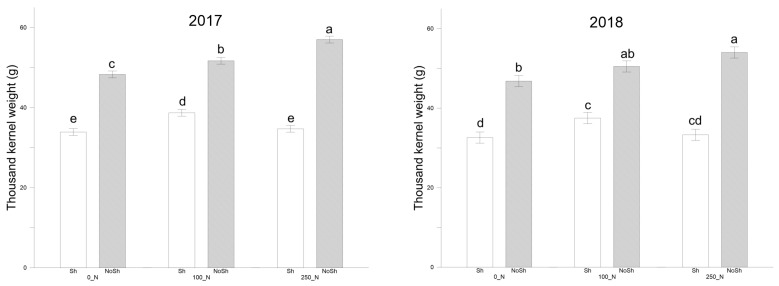
Thousand kernel weight (TKW, g) as recorded for durum wheat at harvest in the 2016–2017 and 2017–2018 cropping seasons. NoSh: 100% PAR; Sh: 20% PAR; 0_N: 0 Kg N ha^−1^; 100_N: 100 Kg N ha^−1^; 250_N: 250 Kg N ha^−1^. Data are averages ± standard errors of n = 3 independent replicates. Different lower case letters indicate significant differences at *p* < 0.05 (Fisher’s LSD test).

**Figure 3:**
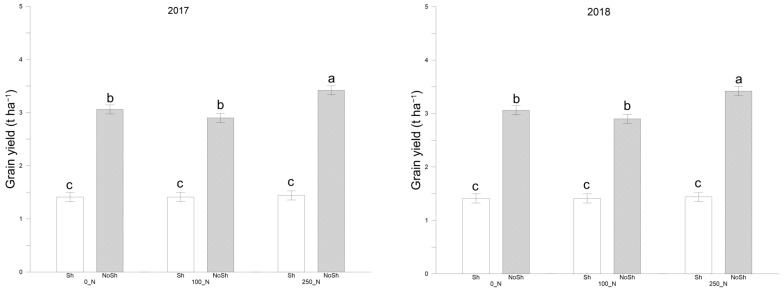
Grain yield (t ha^−1^) as recorded for durum wheat at harvest in the 2016–2017 and 2017–2018 cropping seasons. NoSh: 100% PAR; Sh: 20% PAR; 0_N: 0 Kg N ha^−1^; 100_N: 100 Kg N ha^−1^; 250_N: 250 Kg N ha^−1^. Data are averages ± standard errors of n = 3 independent replicates. Different lower case letters indicate significant differences at *p* < 0.05 (Fisher’s LSD test).

The thousand kernel weight increased significantly in the full-irradiance treatments (Figure 2 and Figure S2). NoSh+250 showed a significant increase in both years compared to all conditions under Sh (*p* < 0.01). NoSh conditions contributed significantly to increasing thousand kernel weight compared to Sh by +46% for 2017 and 2018, respectively. Similarly, for yield production (Figure 3, Figure S2), Sh conditions achieved significantly lower production values (*p* < 0.01) than full-irradiation conditions in both years (Figure 3). The highest values were obtained by the NoSh+250 combination (3.4 t ha^−1^ in 2017 and 2.8 t ha^−1^ in 2018). Nitrogen availability induced significant differences between 250_N and 0_N in 2017 (*p* < 0.05) and between 250_N and the other two doses in 2018 (*p* < 0.05), respectively.

### 2.5. Yield Components

In the 2017 season, ear length was significantly influenced by PAR availability (Table 3, Figure S3). Plants grown under full sunlight exhibited greater ear lengths than those under shading, with overall means of 5.67 cm and 5.08 cm, respectively (*p* < 0.05). Nitrogen rates did not significantly affect ear length, as the overall means across N rates were 5.34 cm for 0_N, 5.50 cm for 100_N, and 5.29 cm for 250_N. No significant interaction between PAR availability and nitrogen rates was observed for ear length. This suggests that while shading reduced ear length, the application of nitrogen did not counteract this effect in 2017. In contrast, the 2018 season showed a significant interaction between PAR availability and nitrogen rates for ear length (*p* < 0.05). Under full sunlight, ear length increased with higher nitrogen rates, reaching 6.42 cm at 100_N and 6.35 cm at 250_N. Under Sh, ear length remained relatively stable across nitrogen rates, with values around 5.50 cm. The overall means for ear length were significantly higher under NoSh than under Sh (6.28 cm and 5.54 cm, respectively) (*p* < 0.05). Nitrogen rates did not have a significant main effect when averaged across PAR treatments, indicating that the positive response to nitrogen was more pronounced under optimal light conditions.

Stem length did not exhibit a significant main effect on PAR availability in either season (Table 3, Figure S3). In 2017, the overall mean was 63.2 cm for full sunlight and 61.2 cm for shading. Similarly, nitrogen rates did not significantly affect stem length, with overall means of 61.7 cm for 0_N, 63.2 cm for 100_N, and 61.8 cm for 250_N. However, a significant interaction between PAR availability and nitrogen rates was found (*p* < 0.05). Under full sunlight, stem length increased with the 100_N rate (66.0 cm), whereas stem length varied less with different nitrogen rates under Sh. This interaction suggests that nitrogen application promoted stem elongation primarily when adequate light was available. In the 2018 season, stem length again showed no significant main effects on PAR availability or nitrogen rates. The overall means were 61.1 cm for NoSh and 58.9 cm for Sh, while nitrogen rates averaged 59.4 cm for 0_N, 61.4 cm for 100_N, and 59.3 cm for 250_N. A significant interaction was present between PAR availability and nitrogen rates (p < 0.05). The highest stem length under full sunlight occurred at the 100_N rate (63.0 cm), whereas stem length remained relatively constant across nitrogen rates under shading. These results indicate that stem elongation responses to nitrogen were influenced by light availability, with notable increases occurring only under full sunlight conditions.

Hectoliter weight, an indicator of grain quality, was significantly affected by both PAR availability and nitrogen rates in both seasons (Table 3, Figure S3). In 2017, hectoliter weight was higher under full sunlight (83.2 kg/hl) compared to shading (81.9 kg/hl) (*p* < 0.05). Nitrogen rates were significantly affected (*p* < 0.01), with overall means of 81.4 kg/hl for 0_N, 83.3 kg/hl for 100_N, and 83.1 kg/hl for 250_N. A significant interaction between PAR availability and nitrogen rates was also observed (*p* < 0.01). Under full sunlight, hectoliter weight increased with higher nitrogen rates, peaking at 84.4 kg/hl for 100_N and 83.9 kg/hl for 250_N. Under Sh, the values were lower and showed less variation with nitrogen application. In 2018, similar patterns were observed for hectoliter weight. Full sunlight conditions resulted in higher values (82.0 kg/hl) than Sh (80.9 kg/hl) (*p* < 0.05). Nitrogen rates again had a significant effect (*p* < 0.01), with overall means of 80.4 kg/hl for 0_N, 81.8 kg/hl for 100_N, and 82.1 kg/hl for 250_N. The interaction between PAR availability and nitrogen rates was significant (*p* < 0.01), indicating that the positive effect of nitrogen on hectoliter weight was more pronounced under full sunlight.

The number of ears per square meter was significantly higher under full sunlight than shading in both seasons (Table 3, Figure S3). In 2017, NoSh had an average of 283.3 ears m^−2^, while Sh plots had 242.6 ears m^−2^ (*p* < 0.05). Nitrogen rates did not significantly affect the number of ears per square meter, with overall means of 260.8 ears m^−2^ for 0_N, 260.5 ears m^−2^ for 100_N, and 267.7 ears m^−2^ for 250_N. This parameter had no significant interaction between PAR availability and nitrogen rates. Similarly, in 2018, full sunlight resulted in 273.1 ears m^−2^ compared to 238.0 ears m^−2^ under shading (*p* < 0.01). Nitrogen rates did not have a significant main effect, and no significant interaction with PAR availability was observed. Overall, shading had a consistent negative impact on key yield components of durum wheat, including ear length, hectoliter weight, and the number of ears per square meter. These effects highlight the importance of adequate light availability for optimal wheat development and yield. Nitrogen fertilizer improved certain yield components, particularly hectoliter weight, under full sunlight conditions. The significant interactions between PAR availability and nitrogen rates for parameters such as ear length, stem length, and hectoliter weight underscore the interplay between light and nitrogen in determining wheat growth responses.

### 2.6. Grain Protein Concentration, Gluten Proteins Content, and Characterization

**Figure 4:**
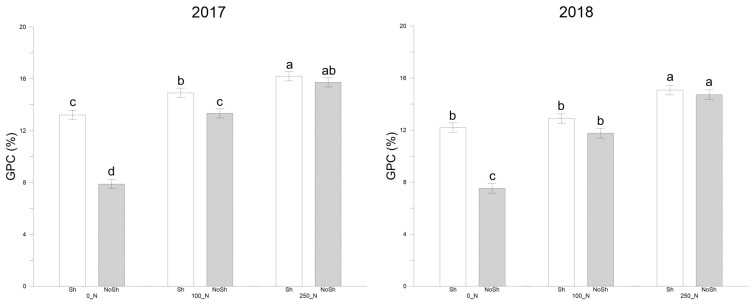
Grain protein content (GPC, %) as recorded for durum wheat at harvest in the 2016–2017 and 2017–2018 cropping seasons. NoSh: 100% PAR; Sh: 20% PAR; 0_N: 0 Kg N ha^−1^; 100_N: 100 Kg N ha^−1^; 250_N: 250 Kg N ha^−1^. Data are averages ± standard errors of n = 3 independent replicates. Different lower case letters indicate significant differences at *p* < 0.05 (Fisher’s LSD test).

GPC values were similar between the two years (Figure 4, Figure S4), with an increasing trend as a function of nitrogen dose. Sh+250N induced higher but not significant values for both years than NoSh+250 (16.20% and 15.73% for 2017; 15.08% and 14.73% for 2018). On average, the Sh condition showed significantly higher values than the NoSh condition (+19.97% for 2017 and +18.08% for 2018, respectively).

Gliadin concentrations showed a significant response to nitrogen treatments, with proportional increases as the dose of N increases (Table 4, Figure S4). The 2017 overall mean indicated higher values for 250_N treatments compared to 0_N, regardless of the irradiation regime. A similar pattern was observed in 2018, with a significant PAR × N interaction highlighting the joint effect of the treatments. In 2017, the concentration of HMW-GS varied significantly with N treatments (*p* < 0.01), reaching a maximum in the 250_N plots. However, the effect of PAR availability was only evident in some conditions, with significantly higher values in NoSh conditions. Similar behavior was seen in 2018, where the 250_N treatment showed the highest value (3.33 mg g^−1^). LMW-GS also responds positively to increasing nitrogen fertilization levels. In 2017, the highest values were observed in the 250_N plots (4.41 mg g^−1^), while the difference between the irradiation treatments was less pronounced. In 2018, the effect of the PAR × N interaction was significant (*p* < 0.01), suggesting an increased sensitivity of this protein fraction to environmental changes. The GS/GLIA ratio, indicative of the balance between glutenin and gliadin fractions, increases significantly with the dose of N. The highest values were recorded for the 250_N treatments in both seasons. Moreover, in 2018, the effect of PAR × N interaction is particularly evident, with an improvement in the ratio in NoSh compared to Sh. Nitrogen application positively affects gluten protein fractions and GS/GLIA ratio, improving gluten quality. PAR availability modulates these effects, highlighting the importance of environmental conditions in determining the final quality of wheat protein.

### 2.7. Starch Content

**Figure 5:**
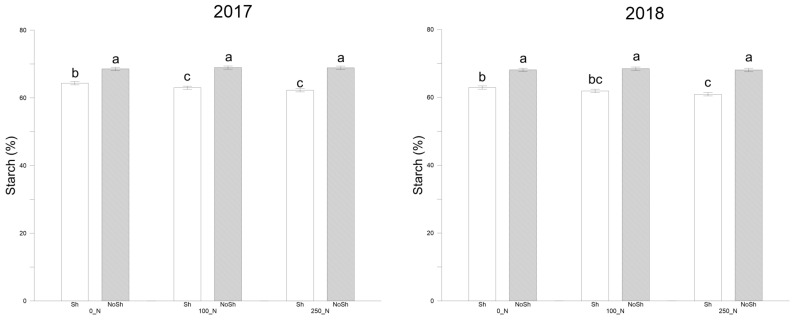
Search content (%) as recorded for durum wheat at harvest in the 2016–2017 and 2017–2018 cropping seasons. NoSh: 100% PAR; Sh: 20% PAR; 0_N: 0 Kg N ha^−1^; 100_N: 100 Kg N ha^−1^; 250_N: 250 Kg N ha^−1^. Data are averages ± standard errors of n = 3 independent replicates. Different lower case letters indicate significant differences at *p* < 0.05 (Fisher’s LSD test).

In the 2017 season, shading conditions significantly influenced the starch percentage in durum wheat grains (Figure 5, Figure S2). Under NoSh conditions, starch content remained consistent across different nitrogen (N) rates: 68.57% for 0_N, 68.96% for 100_N, and 68.91% for 250_N. In contrast, starch content decreased under Sh conditions with increasing nitrogen rates: 64.34%, 63.01%, and 62.27% for 0_N, 100_N, and 250_N, respectively. This indicates significant differences among the treatments under shading, with the highest starch content at the lowest nitrogen rate. The overall means for 2017 showed that starch content was higher under NoSh conditions (68.81%) compared to Sh conditions (63.21%). When averaged across both shading treatments, the differences in starch content among nitrogen rates were minimal, 66.46% for 0_N, 65.99% for 100_N, and 65.59% for 250_N, suggesting that nitrogen application had a limited effect on starch content in NoSh conditions. In 2018, a similar pattern was observed. Starch content remained significantly higher under NoSh conditions than Sh conditions, with overall means of 68.23% and 61.91%, respectively. Under NoSh conditions, starch content was high and relatively stable across nitrogen rates: 68.12% for 0_N, 68.47% for 100_N, and 68.09% for 250_N. Under Sh conditions, starch content decreased with higher nitrogen rates, 62.92% for 0_N, 61.90% for 100_N, and 60.91% for 250_N, indicating significant differences among the treatments under shading. The overall means for nitrogen rates in 2018 showed a slight decline in starch content with increasing nitrogen application: 65.52% for 0_N, 65.19% for 100_N, and 64.60% for 250_N. As in 2017, these differences were not statistically significant when averaged over both shading treatments, highlighting that shading had a more pronounced effect on starch content than nitrogen rates.

## 3. Discussion

### 3.1. Meteorological Data

The results of our experiment showed how PAR availability during flowering and nitrogen fertilization interact to significantly influence the productivity and quality of durum wheat under Mediterranean conditions. Aridity during key phenological phases significantly affected yield parameters in our study. In the 2016–2017 season, abundant rainfall during the vegetative phase (mid-January to end of April), especially in January and February, likely enhanced seedling emergence and tiller development. This resulted in more ears per square meter, as sufficient soil moisture during early growth promotes spike formation [27]. Conversely, the 2017–2018 season experienced lower rainfall during the vegetative phase but received substantial precipitation during the reproductive phase (May and June). This increased moisture likely improved grain filling and ear length, as adequate water supports biomass accumulation and grain development during this stage. Arid conditions during grain filling in 2016–2017 may have accelerated plant senescence and limited nutrient translocation to grains, affecting their weight and quality. This could explain the higher hectoliter weight observed, as reduced moisture can concentrate grain constituents. In contrast, higher rainfall during grain filling in 2017–2018 may have diluted grain protein content, reducing hectoliter weight due to increased carbohydrate accumulation.

### 3.2. Physiological Traits

Shading increased chlorophyll and carotenoid levels, suggesting an adaptive plant response but impaired spike formation and seed filling, thus reducing yield. Shading stimulates compensatory chlorophyll synthesis and offsets reduced light availability by increasing chlorophyll levels to support essential metabolic functions [28]. Applying additional nitrogen fertilizer enhances the capacity of wheat leaves to capture light energy, improves the efficiency of light energy conversion, and enables more effective use of the captured light energy for photosynthesis [11]. As reported by Tincai et al. [29], nitrogen (N_2_) helped improve ΦPSII and qP and reduced NPQ in wheat under no-shade and mild-shade conditions. Zhang et al. [30] argued that the regulatory effect of nitrogen fertilizer on qP and NPQ in shading is higher than in normal light. Still, its regulatory effect on ΦPSII is lower than that in normal light. The study highlighted the critical impact of PAR availability and nitrogen fertilization on durum wheat growth, physiology, and grain quality. In addition, the interaction between PAR and nitrogen was significant for the other physiological traits. The highest NDVI, SPAD, and total chlorophyll content values were recorded under shading conditions with high nitrogen fertilization (Sh+250_N). This suggests that adequate nitrogen supply can mitigate the negative effects of shading.

### 3.3. Grain Yield

Our findings suggest that shading significantly reduces key yield components of durum wheat, including ear length, hectoliter weight, and the number of ears per square meter. In the 2017 season, plants grown under full sunlight exhibited greater ear lengths than those under shading (5.67 cm vs. 5.08 cm, respectively). A similar trend was observed in 2018, with ear lengths of 6.28 cm under full sunlight and 5.54 cm under shading. These findings are consistent with [23], who reported that shading during stem elongation reduces spike dry weight at anthesis and grain number due to decreased assimilate supply from reduced photosynthesis. Shading limits photosynthetic capacity by decreasing light availability, which is crucial during the critical periods of stem elongation and early reproductive development. This reduction in photosynthesis leads to insufficient assimilate production for optimal spike and grain development [2,25]. In our study, the number of ears per square meter was significantly higher under full sunlight than shading in both seasons, reinforcing the idea that adequate light is essential for ear formation and development. Nitrogen application showed variable effects on yield components depending on light conditions. In 2017, nitrogen rates did not significantly affect ear length or stem length, and no significant interaction with PAR availability was observed. However, in 2018, a significant interaction between PAR availability and nitrogen rates was found for ear length. Under full sunlight, ear length increased with higher nitrogen rates, reaching up to 6.42 cm at 100_N and 6.35 cm at 250_N, while under shading, ear length remained relatively stable. This suggests that nitrogen promotes growth more effectively when light conditions are optimal, aligning with findings by Mu et al. [2] that compensatory mechanisms under shading are insufficient to overcome reduced photosynthetic capacity. Hectoliter weight, an indicator of grain quality, was significantly affected by both PAR availability and nitrogen rates in both seasons. The positive effect of nitrogen on hectoliter weight was more pronounced under full sunlight, indicating that adequate light enhances the plant’s ability to utilize additional nitrogen for grain filling [25]. It was observed that shading during early reproductive development impairs photosynthesis, leading to reduced grain number and lower grain yield, which supports our findings on the importance of light in conjunction with nutrient availability. Stem length exhibited a significant interaction between PAR availability and nitrogen rates. Under full sunlight, stem length increased with nitrogen application, particularly at the 100_N rate, whereas under shading, stem length remained relatively constant across nitrogen rates. This indicates that nitrogen promotes stem elongation primarily when sufficient light is available, possibly due to enhanced photosynthetic activity and assimilate allocation under optimal light conditions. Terrile et al. [23] also noted that wheat adjusts growth efficiency at the spike level in response to shading, which can affect stem and spike development. Multiple studies have shown that shading stress causes yield loss. This is mainly caused by grain number and weight reduction [24,31]. According to Retkute et al. [32], the impact of reduced radiation on final yield depends on the phenological stage during which shading occurs and its duration, with variations affecting yield components differently. When shading is applied during the pre-flowering stage (e.g., for ~30 days), the final yield is primarily influenced by a reduction in the number of grains per ear and per unit area [32,33]. In contrast, shading imposed later, after flowering, predominantly reduces the number of grains per square meter and grain weight [34].

### 3.4. Grain Protein and Gluten Fractions

Our results suggest that N fertilization may be used to improve protein and gluten concentration in durum wheat production. These findings are consistent with those reported in previously published studies [35,36]. The results show that N application significantly increases the concentration of gluten protein fractions, with a clear progression from 0_N to 250_N treatments. This phenomenon can be explained by the stimulating effect of nitrogen on protein synthesis in wheat plants, which has already been reported by previous studies [37]. Gliadins, in particular, show a marked response, highlighting their primary role as reserve proteins.

In contrast, glutenins, both HMW-GS and LMW-GS, respond more markedly to high doses of N, indicating that nitrogen availability also influences gluten quality in terms of structure and functionality [37]. These results confirm that the optimal management of nitrogen fertilization is essential to maximize the technological quality of flour. However, high doses of N could have environmental implications and increase production costs, making it necessary to strike a balance between agronomic performance and sustainability. The analysis shows that PAR availability modifies the response of protein fractions, albeit with varying intensity. NoSh conditions are generally associated with higher protein concentration, especially for glutenins (HMW-GS and LMW-GS). This result is consistent with the positive effect of light on photosynthesis and, consequently, carbon availability for protein synthesis.

In contrast, in plots under Sh, PAR reduction limited the synthesis of some protein fractions, probably due to lower photosynthetic efficiency. This phenomenon suggests that gluten protein fractions are sensitive to irradiation conditions during key stages of caryopsis development, such as protein accumulation in the post-flowering stage [38]. The interaction between PAR and N for most of the parameters analyzed indicates that the two factors act synergistically. Under NoSh, increased nitrogen fertilization amplifies the benefits on protein content, reaching maximum levels with the 250_N treatment. However, under Sh, the effect of nitrogen is attenuated, highlighting the importance of radiation in maximizing the efficiency of applied nitrogen. The GS/GLIA ratio, an indicator of gluten quality, increases significantly with increasing N doses and under full sun conditions. This suggests that the technological quality of flour is improved by nitrogen application, which promotes greater synthesis of glutenins than gliadins [39]. However, differences between seasons (2017 and 2018) indicate that climatic factors may influence the response. For example, high temperatures during the caryopsis filling stage may have reduced protein accumulation under some conditions [40].

### 3.5. Starch

The results from both the 2017 and 2018 seasons demonstrate that shading significantly negatively impacts starch accumulation in durum wheat grains. Under NoSh conditions, starch percentages were consistently high, averaging around 68% in both years. This suggests that adequate light availability allows plants to maximize photosynthesis and carbohydrate synthesis, leading to optimal starch accumulation in the grains. Similar findings were reported by Yang et al. [41], who observed that shading reduced starch content by decreasing the photosynthetic efficiency of flag leaves, resulting in less carbohydrate synthesis and a lower grain-filling rate. Under NoSh conditions, varying nitrogen rates did not significantly affect starch content; percentages remained high and showed minimal variation across the 0_N, 100_N, and 250_N treatments. This indicates that increasing nitrogen rates does not enhance or diminish starch synthesis when light is sufficient. Plants likely have adequate resources to support both vegetative growth and starch accumulation without being limited by nitrogen availability.

In contrast, an inverse relationship between nitrogen rates and starch content was observed under Sh. In both years, the highest starch percentages under shading were recorded at the 0_N rate, with a progressive decline as nitrogen rates increased to 100_N and 250_N. These decreases were statistically significant, suggesting that higher nitrogen availability may shift the plant’s metabolic priorities toward vegetative growth under limited light conditions at the expense of starch accumulation in the grains. Wen [42] reported that shading reduced starch accumulation by decreasing the activities of enzymes involved in starch synthesis and reducing substrate availability. Specifically, early shading limited substrates, while mid-stage shading further reduced starch synthase activities, leading to reduced overall starch content.

Additionally, Li et al. [3] found that shading reduced grain starch concentration by limiting photosynthesis and substrate availability, causing a significant reduction in smaller B-type starch granules while increasing the proportion of larger A-type granules. This shift indicates a prioritization of specific metabolic pathways under shading conditions, which may explain the decreased starch content observed at higher nitrogen rates under shading in our study. Judel and Mengel [43] observed that shading reduced the production and storage of non-structural carbohydrates in culms and leaves, directly affecting their mobilization to the grain during the filling period and limiting grain weight and yield. These findings reinforce the critical role of light availability in starch synthesis and accumulation. No significant interactions between shading and nitrogen rates were observed for starch content in either year. This indicates that while shading and nitrogen independently affect starch accumulation, their effects do not interact synergistically or antagonistically. Shading consistently reduced starch content regardless of the nitrogen rate applied, highlighting the overriding influence of light availability on starch accumulation.

### 3.6. General Discussion

The decision to shade flowering durum wheat at 80% was motivated by the need to simulate extreme light stress conditions. This, in nature, could occur due to events such as pollution, intense cloud cover, or other factors that can drastically reduce light availability [44]. Intense shading represented a limit test to understand the physiological limits of the crop and its ability to adapt or compensate in the Mediterranean environment. Moreover, global warming, changes in rainfall patterns, and increases in the frequency and intensity of extreme events are already affecting agricultural systems and food production [45]. These changes are measurable through direct (variation in the length of the growth cycle, crop yield, and quality of the products) and indirect impacts (variation in available water resources, distribution, and the extent of pest and plant diseases). The effects on the agricultural sector vary between geographical regions and local environmental conditions, and also depend on the sector’s sensitivity, adaptive capacity, and degree of exposure to climate hazards [46].

This study showed how light availability and fertilization practices affect durum wheat yield as well as quality. This is in line with projections by the International Food Policy Research Institute, which indicate how rising temperatures, variability in rainfall, and extreme weather events can affect production, emphasizing the importance of adopting sustainable agricultural practices to mitigate adverse effects and ensure food security [47]. Moreover, improving nitrogen use efficiency (NUE) will be critical in reducing excessive fertilizer inputs, which contribute to greenhouse gas emissions and eutrophication. By maximizing crop uptake and minimizing losses, higher NUE will promote sustainable agricultural systems that are more resilient under changing climatic conditions [48].

Under Sh conditions, grain yield responded only to a limited extent to an increasing nitrogen supply. This result indicates that higher nitrogen applications under reduced light availability would probably not lead to significant yield increases. Consequently, excessive N fertilization under these conditions could be inefficient both from an economic point of view (fertilizer costs) and from an ecological point of view (risk of leaching). In contrast, yields under full sunlight (NoSh) showed a more pronounced response to higher amounts of nitrogen. Under such conditions, higher N doses (within sustainable thresholds) may be beneficial to maximize productivity, provided that other factors are carefully weighed, including fertilizer costs, pedoclimatic conditions (e.g., water availability, soil properties), and applicable environmental regulations (e.g., nitrogen application limits, restricted fertility periods). Therefore, nitrogen fertilization strategies could also take into account the available radiation in the respective growing area, which is influenced by factors such as latitude, day length, light intensity, and shading by the local orography [49,50,51].

## 4. Materials and Methods

### 4.1. Site Description

Field experiments were conducted in Teramo (Mosciano Sant’Angelo, Italy, 42°42′ N, 13°52′ E, 101 m a.s.l.) over two cropping seasons, 2016–2017 and 2017–2018, hereafter referred to as 2017 and 2018, respectively. The area experiences a typical Mediterranean climate, with an average annual rainfall of 732 mm, primarily occurring between October and April, based on data collected over 58 years. The average maximum temperatures range from 11 °C to 29 °C, while the average minimum temperatures range from 2 °C to 17 °C. The soil is a typical medium-clay with the following characteristics: 23% sand, 34% silt, 43% clay, 1.0% organic matter, a pH of 7.9, and 1.0% total nitrogen. Meteorological data were recorded at a weather station approximately 1 km from the experimental field.

### 4.2. Agronomic Management and Experimental Design

The experiment was aimed at evaluating different levels of photosynthetically active radiation (PAR) (µmol m^−2^ s^−1^) availability and N fertilization rates in durum wheat (*Triticum turgidum* L. subsp. *Durum* (Desf); six experimental treatments were compared. Conventional agricultural management was used, with the soil subjected to moldboard plowing (complete soil inversion) to a depth of 25 cm during the summer. In both growing seasons, the preceding crop was the faba bean (*Vicia faba* L. var. *minor*). The durum wheat cultivar was “Saragolla”, and the faba bean cultivar was “Protabath”. Durum wheat was sown on 23 November in 2016 and 9 December and 2017, respectively, with a direct seeder (Gaspardo Direttissima, Gruppo Maschio Gaspardo S.p.A., Campodarsego, PD, Italy) at a seeding rate of 350 seeds m^−2^. In both years, a fungicide application was carried out at the beginning of anthesis using Sphere (Trifloxystrobin and Ciproconazole, Bayer Crop Science Italia, Borgo Sabotino, LT, Italy) at a rate of 1 L ha^−1^. No herbicide treatments were applied. The experimental design consisted in a split-plot arranged on randomized complete blocks with three replications. Two levels of PAR represented the main plots. They consisted of a fully sunny (unshaded) treatment (100% of PAR availability, NoSh) and a shaded treatment (20% of PAR availability, Sh), and three levels of N rates made up the sub-plots (0 kg N ha^–1^, 0_N; 100 kg N ha^–1^, 100_N; and 250 kg N ha^–1^, 250_N). Nitrogen was applied as urea in a single application on 22 March 2017 and 7 April 2018, at the phenological stage of stem elongation. Each plot measured 3.90 m^2^ (1.30 × 3 m). Shading was achieved using a green shading net (supplied by CARRETTA Tessitura s.n.c., Carrè, VI, Italy), which was mounted on a rigid, removable structure positioned above the vegetation to block incoming light from both the top and sides. Green shading nets were applied starting from anthesis (27 April and 3 May for 2017 and 2018, respectively), Zadoks Decimal code 61 [52]. The PAR intensity was measured every 15 min and the % of shading was determined by comparing the average PAR values under the net with the average PAR values in the open field. Under the green net, the percentage of reduction with respect to outdoor conditions amounted to 80%. The mean temperatures recorded during the crop cycle were 16.25 °C and 15.75 °C, respectively, in NoSh and Sh in 2017 and 19.9 °C and 19.1 °C, respectively, in NoSh and Sh in 2018. Slight differences were also obtained for soil relative humidity (RH), in both growing seasons; the highest values were registered in plants under the shading net (13.9% vs. 15.7% and 9% vs. 12.9%, respectively, in 2017 and 2018); on the other hand, air RH was similar under the two conditions (on average 65.6% and 74% in 2017 and 2018, respectively).

### 4.3. Physiological Traits

Canopy reflectance for each experimental unit was measured using a Spectroradiometer (Handheld 2 Pro Portable Field Spec, ADS Inc., Boulder, CO, USA), and the normalized difference vegetation index (NDVI) was subsequently calculated as described by Rouse et al. [53].

(1) $$NDVI670\frac{\left(R800-R670\right)}{\left(R800+R670\right)}$$

Chlorophyll content was estimated using an SPAD (Soil–Plant Analysis Development) meter (502 Plus portable chlorophyll meter, Konica Minolta, Inc., Tokyo, Japan). Multiple readings were taken on the mid-sections of 10 fully expanded leaves per experimental unit across five observation dates, ranging from anthesis to the early dough stages (DC61, DC69, DC71, DC75, and DC83). The measurements focused on the flag leaf or, when not available, the greenest leaves [54].

### 4.4. Chlorophyll and Carotenoid Contents

Ten days after anthesis, chlorophyll a (Chla), chlorophyll b (Chlb), and carotenoid (Car) contents in flag leaves were determined according to Arnon et al. [55] and Djebaili et al. [56]. Sampled leaves were washed using deionized water; two cyclic leaflets (10 mm diameter) were obtained from one sample for each experimental unit. Pigments were extracted from fresh leaf sub-samples using an 80% acetone solution. The extracts were then read at 645 and 663 nm for Chla and Chlb, respectively, and at 470 Nm for Car using a spectrophotometer (Jenway 6300, Jenway, Stone, UK). The pigment contents, expressed as µg mg^−1^ FW, were then calculated as follows:

Chla = [(12.7) × (Abs_663_)] − [(2.69) × (Abs_645_) × V/1000 × FW] (2)

Chlb = [(22.9) × (Abs_645_)] − [(4.68) × (Abs_663_) × V/1000 × FW] (3)

Car = [(1000) × (Abs_470_)] − (1.82 Chla) − (85.02 Chlb) × V/198 × 1000 × FW] (4)

where Abs is absorbance, V is final extraction volume, and FW is the sample’s fresh weight.

### 4.5. Yield and Merceological Parameters

At physiological maturity (22 June and 28 June for 2017 and 2018, respectively), one square meter of wheat plants was manually harvested from each experimental plot to assess yield components and various commercial and technological quality parameters. Grain yield was calculated at 13% moisture content, and the number of ears per unit area was determined by counting spikes within the one-square-meter plot. Additionally, twenty spikes per plot were collected to measure ear length and plant height, with height measured from the soil to the top of the spike. The 1000-kernel weight (TKW, g 1000^−1^ seeds) and hectoliter weight (HW, kg hL^−1^) were measured by ISO 7971-1 (2009) and ISO 520 (2010) [57,58].

### 4.6. Grain Quality Analysis

Grain sub-samples from each window plot were milled into a fine powder using a Knifetec TM 1095 mill (Foss, Hillerød, Denmark), and the resulting whole-grain flour was used to analyze various quality-related parameters. Grain nitrogen content was measured using the standard Kjeldahl method, and grain protein concentration (GPC, %) was calculated by multiplying the nitrogen content by 5.7 [59]. The analysis of gluten proteins was determined, as reported by Pagnani et al. [60]. The starch content in whole grain flour (ST; %) was determined using a Total Starch Assay Kit (K-TSTA, Megazyme, Irishtown, Ireland) as reported in Cammerata et al. [61].

### 4.7. Statistical Analysis

A two-way analysis of variance (ANOVA), according to a split-plot design arranged on a randomized complete block, was performed with the free Excel plugin DSAASTAT^®^ VBA macro, version 1.1 [62]. The separation of the means was set at a 1% and 5% (*p* < 0.01 and *p* < 0.05, respectively) level of significance by Fisher’s least significant difference (LSD) test. Prior to ANOVA, data were analyzed to test the normality and homoscedasticity assumptions.

## 5. Conclusions

The interaction between photosynthetically active radiation and nitrogen fertilization plays a significant role in the productivity and quality of durum wheat under Mediterranean conditions. Full sunlight treatments have led to a significant increase in grain yield, thousand kernel weight, and hectoliter weight compared with Sh. The availability of nitrogen further improved these parameters, especially under optimal light conditions, with the highest values observed at the 250 kg N ha^−1^ rate. Chlorophyll content, SPAD, and NDVI followed similar trends, showing higher concentrations and values with higher nitrogen rates, especially under full sunlight. Shading conditions led to higher chlorophyll and carotenoid content but reduced grain yield, resulting in fewer ears per square meter, a reduced length of the ears, and reduced protein content. The impact of shading on wheat growth was most pronounced during the reproductive stages, with the application of nitrogen having a positive effect on the protein content of the wheat and on the protein fractions of the gluten. Precipitation patterns in the two seasons have further contributed to variability in yield and quality, with the 2016–2017 season benefiting from higher rainfall during vegetative growth and the 2017–2018 season having more favorable conditions during grain filling. These results underline the importance of optimizing both light availability and nitrogen management to improve the yield and quality of durum wheat under Mediterranean conditions, especially in regions where abiotic stress factors such as heat and drought prevail. The positive effect of nitrogen on wheat quality, particularly on protein content and gluten protein fractions, is a crucial aspect for improving the quality of the final product, which could have practical applications for durum wheat producers. Furthermore, the results stress the importance of considering environmental variables like shading conditions and seasonal rainfall, which significantly impact wheat growth and development. The fact that shading has shown negative effects on yield but not on chlorophyll content suggests that optimal light is a key factor in maximizing productivity. Integrating management strategies combining adequate lighting with nitrogen fertilization practices could, therefore, be crucial to improving sustainability and production efficiency in Mediterranean areas. Finally, the analysis of precipitation patterns in the two seasons offers an additional dimension to the research, showing how climatic factors can influence wheat yield and quality.

Although the results of this study were satisfactory, it would be optimal to extend the research by increasing the number of cycles to assess the long-term sustainability of these practices and determine the impact on yield and quality. For further development, it may be necessary to evaluate more wheat varieties to highlight differences in responses to light and nitrogen management practices and to observe which varieties are more resilient to expected environmental stresses. It would also be interesting to include a wider range of stress variables and their combinations to assess their impact on the crop in terms of yield and quality.

Further development could be achieved by initiating more mechanically oriented studies of physiological processes. A detailed analysis of chlorophyll and carotenoid composition, which then translates into variations in grain yield and quality, could lead to a deeper understanding of the key mechanisms regulating plant growth and development. This approach would not only allow a better correlation of physiological data with agronomic parameters, but also identify critical points in plant assimilation processes and metabolism and thus improve agronomic management strategies.

## Figures and Tables

**Table 1:** Chlorophyll and carotenoid contents (mg g^−1^ FW) in flag leaves of durum wheat plants subjected to two levels of photosynthetically active radiation (PAR) availability (NoSh: full sunlight; Sh: plots shaded by a green shading net) and three different nitrogen (N) application rates (100_N: 100 kg ha^−1^ of N; 250_N: 250 kg ha^−1^ of N; and 0_N: zero nitrogen application) in two growing seasons, 2017 and 2018, 10 days after anthesis. Data are averages of n = 3 independent replicates. Means followed by different letters (upper case letters: main effects; lower case letters: effects of interaction) significantly differ according to Fisher’s LSD test (*p* < 0.05).

Year	Treatments ^a^	Chl a			Chl b			Chl tot			Car		
		Sh	NoSh	*O. M.* ^b^	Sh	NoSh	*O. M.*	Sh	NoSh	*O. M.*	Sh	NoSh	*O. M.*
2017	0_N	4.41 c	2.70 d	3.56 C	2.05 b	0.98 c	1.52 B	6.46 c	3.68 c	5.07 C	0.36	0.52	0.44 B
	100_N	7.08 a	3.18 d	5.13 B	2.78 ab	0.97 c	1.88 AB	9.86 ab	4.15 c	7.01 B	0.39	0.60	0.49 B
	250_N	8.11 a	5.85 b	6.98 A	2.94 a	2.75 ab	2.84 A	11.05 a	8.60 b	9.82 A	0.64	0.89	0.77 A
	*O. M.*	6.54 A	3.91 B		2.59 A	1.57 B		9.13 A	5.48 B		0.46 B	0.67 A	
PAR availability		**			*			**			**		
N rates		**			**			**			**		
PAR × N		**			*			**			n.s.		
2018	0_N	3.26 d	1.79 e	2.53 C	0.94 c	0.64 d	0.79 B	4.20	2.43	3.32 C	0.28 e	0.39 bc	0.33 B
	100_N	5.83 a	4.74 b	5.29 A	2.00 a	1.15 bc	1.58 A	7.83	5.90	6.86 A	0.37 cd	0.46 b	0.41 A
	250_N	4.31 bc	4.00 c	4.15 B	1.31 b	0.92 c	1.12 B	5.62	4.92	5.27 B	0.31 de	0.54 a	0.43 A
	*O.M.*	4.47	3.51		1.42 A	0.91 B		5.89 A	4.42 B		0.32 B	0.46 A	
PAR availability		n.s.			**			*			*		
N rates		**			**			**			*		
PAR × N		*			*			n.s.			*		

^a^ Treatments: NoSh: 100% PAR; Sh: 20% PAR; 0_N: 0 Kg N ha^−1^; 100_N: 100 Kg N ha^−1^; 250_N: 250 Kg N ha^−1^. ^b^ Overall means. * *p* < 0.05; ** *p* < 0.01; n.s. = not-significant. Degrees of freedom: PAR availability, one; N rates, two; PAR availability × N rates, two; residual, eight.

**Table 2:** SPAD (soil plant analysis development) and NDVI (normalized difference vegetation index) as measured in levels of photosynthetically active radiation (PAR) availability (NoSh: full sunlight; Sh: plots shaded by a green shading net) in durum wheat plants subjected to three nitrogen (N) application rates (100_N: 100 kg ha^−1^ of N; 250_N: 250 kg ha^−1^ of N; and 0_N: zero nitrogen application) in 2017 and 2018. SPAD and NDVI: average seasonal values (from anthesis to early dough). Data are averages of n = 3 independent replicates Means followed by different letters (upper case letters: main effects; lower case letters: effects of interaction) significantly differ according to Fisher’s LSD test (*p* < 0.05).

SPAD						
	2017			2018		
**Treatments** **^a^**	Sh	NoSh	*O. M.* ^b^	Sh	NoSh	*O. M.* ^b^
0_N	48.7 ab	40.4 d	44.5 B	46.4 bc	38.4 e	42.4 B
100_N	50.4 a	44.3 c	47.4 A	48.3 ab	42.5 d	45.4 AB
250_N	49.3 a	46.4 bc	47.9 A	48.7 a	45.5 c	47.1 A
*O. M.* ^b^	49.5 A	43.7 B		47.8 A	42.2 B	
PAR availability		**			*	
N rates		**			**	
PAR × N		**			*	
**NDVI**						
	2017			2018		
**Treatments** **^a^**	Sh	NoSh	*O. M.* ^b^	Sh	NoSh	*O. M.* ^b^
0_N	0.879 a	0.789 c	0.834 B	0.849	0.790	0.820 B
100_N	0.879 a	0.848 b	0.864 A	0.863	0.856	0.859 A
250_N	0.894 a	0.840 b	0.867 A	0.883	0.871	0.877 A
*O. M.* ^b^	0.884 A	0.826 B		0.865	0.839	
PAR availability		**			n.s.	
N rates		**			**	
PAR × N		**			**	

^a^ Treatments: NoSh: 100% PAR; Sh: 20% PAR; 0_N: 0 Kg N ha^−1^; 100_N: 100 Kg N ha^−1^; 250_N: 250 Kg N ha^−1^. ^b^ Overall means. * *p* < 0.05; ** *p* < 0.01; n.s. = not-significant. Degrees of freedom: PAR availability, one; N rates, two; PAR availability x N rates, two; residual, eight.

**Table 3:** Shading effect on yield components of durum wheat plants subjected to two levels of photosynthetically active radiation (PAR) availability (NoSh: full sunlight; Sh: plots shaded by a green shading net) and three different nitrogen (N) application rates (100_N: 100 kg ha^−1^ of N; 250_N: 250 kg ha^−1^ of N; and 0_N: zero nitrogen application) in two growing seasons, 2017 and 2018. Data are averages of n = 3 independent replicates. Means followed by different letters (upper case letters: main effects; lower case letters: effects of interaction) significantly differ according to Fisher’s LSD test (*p* < 0.05).

Year	Treatments ^a^	Ear Length (cm)			Stem Length (cm)			Hectolitre Weight (kg/hl)			Ears (num m^−2^)		
		Sh	NoSh	*O. M.* ^b^	Sh	NoSh	*O. M.*	Sh	NoSh	*O. M.*	Sh	NoSh	*O. M.*
2017	0_N	4.98	5.71	5.34	60.4	63.0	61.7	81.4	81.3	81.3 B	243.5	278.0	260.8
	100_N	5.33	5.68	5.50	60.4	66.0	63.2	82.1	84.4	83.3 A	234.5	286.5	260.5
	250_N	4.95	5.62	5.29	62.8	60.8	61.8	82.3	83.9	83.1 A	249.8	285.5	267.7
	*O. M.*	5.08 B	5.67 A		61.2	63.2		81.9 B	83.2 A		242.6 B	283.3 A	
PAR availability		*			n.s.			*			*		
N rates		n.s.			n.s.			**			n.s.		
PAR × N		n.s.			*			**			n.s.		
2018	0_N	5.63 c	6.07 b	5.85	57.7 d	61.1 ab	59.4	80.3 c	80.5 c	80.4 B	235.7	265.2	250.4
	100_N	5.48 c	6.42 a	5.95	59.8 cd	63.0 a	59.3	80.9 bc	82.7 a	81.8 A	240.4	274.5	257.5
	250_N	5.50 c	6.35 a	5.93	59.2 cd	59.3 bc	61.4	81.4 b	82.9 a	82.1 A	238.0	279.7	258.8
	*O. M.*	5.54 B	6.28 A		58.9	61.1		80.9 B	82.0 A		238.0 B	273.1 A	
PAR availability		*			n.s.			*			**		
N rates		n.s.			n.s.			**			n.s.		
PAR × N		*			*			**			n.s.		

^a^ Treatments: NoSh: 100% PAR; Sh: 20% PAR; 0_N: 0 Kg N ha^−1^; 100_N: 100 Kg N ha^−1^; 250_N: 250 Kg N ha^−1^. ^b^ Overall means. * *p* < 0.05; ** *p* < 0.01; n.s. = not-significant. Degrees of freedom: PAR availability, one; N rates, two; PAR availability × N rates, two; residual, eight.

**Table 4:** Gluten fractions (mg g^−1^ flour) (gliadins; HMW-GS: high molecular weight glutenins; LMW-GS: low molecular weight glutenins; total GS/GLIA: HMW-GS + LMW-GS) and their ratios as recorded at harvest in the 2017 and 2018 cropping seasons. Treatments: two levels of photosynthetically active radiation (PAR) availability (NoSh: full sunlight; Sh: plots shaded by a green shading net) and three different nitrogen (N) application rates (100_N: 100 kg ha^−1^ of N; 250_N: 250 kg ha^−1^ of N; and 0_N: zero nitrogen application). Data are averages of n = 3 independent replicates. Means followed by different letters (upper case letters: main effects; lower case letters: effects of interaction) significantly differ according to Fisher’s LSD test (*p* < 0.05).

Year	Treatments ^a^	Gliadins			HMW-GS			LMW-GS			Total GS/GLIA		
		Sh	NoSh	*O. M.* ^b^	Sh	NoSh	*O. M.*	Sh	NoSh	*O. M.*	Sh	NoSh	*O. M.*
2017	0_N	13.41	12.41	12.91 B	2.42	0.97 d	1.70 C	3.65	1.24	2.45 B	19.49	14.62	17.06 B
	100_N	14.06	12.98	13.52 AB	3.44 bc	2.99 cd	3.21 B	4.16 a	3.53	3.85 A	21.66	19.50	20.58 A
	250_N	14.60	13.53	14.07 A	3.82 ab	4.22 a	4.02 A	4.41 a	3.83	4.12 A	22.84	21.58	22.21 A
	*O. M.*	14.03	12.97		3.23 A	2.73 B		4.07 A	2.87 B		21.3 A	18.5 B	
PAR availability		n.s.			*			**			**		
N rates		*			**			**			**		
PAR × N		n.s.			**			**			**		
2018	0_N	12.87	11.77	12.32 c	1.89 c	1.00 d	1.45 b	3.04	0.87	1.96 B	17.80 b	13.65 c	15.73 C
	100_N	13.62	12.26	12.94 b	2.89 b	2.35 c	2.62 a	3.43	2.44	2.93 A	19.94 a	17.05 b	18.49 B
	250_N	13.94	12.87	13.41 a	3.17 ab	3.49 a	3.33 a	3.70	3.05	3.37 A	20.81 a	19.41 a	20.11 A
	*O. M.*	13.48 a	12.30 b		2.65	2.28		3.39 A	2.12 B		19.52 A	16.70 B	
PAR availability		*			n.s.			**			**		
N rates		**			**			**			**		
PAR × N		n.s.			*			**			**		

^a^ Treatments: NoSh: 100% PAR; Sh: 20% PAR; 0_N: 0 Kg N ha^−1^; 100_N: 100 Kg N ha^−1^; 250_N: 250 Kg N ha^−1^. ^b^ Overall means. * *p* < 0.05; ** *p* < 0.01; n.s. = not-significant. Degrees of freedom: PAR availability, one; N rates, two; PAR availability × N rates, two; residual, eight.

## Data Availability

Data are contained within the article and Supplementary Materials.

## Supplementary Materials

The following supporting information can be downloaded at https://www.mdpi.com/article/10.3390/plants14010076/s1. Figure S1. Interactive effects of photosynthetically active radiation (PAR) level and nitrogen application rate (N, kg ha^−1^) on chlorophyll (Chl a, Chl b, and total chlorophyll, mg g^−1^ FW) and carotenoid (Car, mg g^−1^ FW) content during two growing seasons (2017 and 2018). Yellow lines represent NoSh (no shading), and grey lines represent Sh (shading). Figure S2. Interactive effects of photosynthetically active radiation (PAR) availability and nitrogen application rate (N, kg ha^−1^) on grain yield (t ha^-1^), thousand kernel weight (g) and starch content (%) during two growing seasons (2017 and 2018). Yellow lines represent NoSh (no shading), and grey lines represent Sh (shading). Figure S3. Interactive effects of photosynthetically active radiation (PAR) availability and nitrogen application rate (N, kg ha^−1^) on yield components in two growing seasons (2017 and 2018), showing ear length (cm), stem length (cm), hectoliter weight (kg/hl) and ear density (num m^−2^). Yellow lines represent NoSh (no shading), and grey lines represent Sh (shading). Figure S4. Interactive effects of photosynthetically active radiation (PAR) availability and nitrogen application rate (N, kg ha^-1^) on grain protein concentration (GPC, %), gluten protein content (gliadins, HMW-GS, and LMW-GS, mg g^-1^ flour), and GS/GLIA ratio in two growing seasons (2017 and 2018). Yellow lines represent NoSh (no shading), and grey lines represent Sh (shading).
