# Supplementary material for: Effect of Flowering Shading on Grain Yield and Quality of Durum Wheat in a Mediterranean Environment
Source: Plants (Basel). 2024 Dec 29;14(1):76. doi: 10.3390/plants14010076 (PMC11722777; doi:10.3390/plants14010076)
Supplement: Supplementary file 1 [file plants-14-00076-s001.zip › plants-3386955-supplementary.pdf]

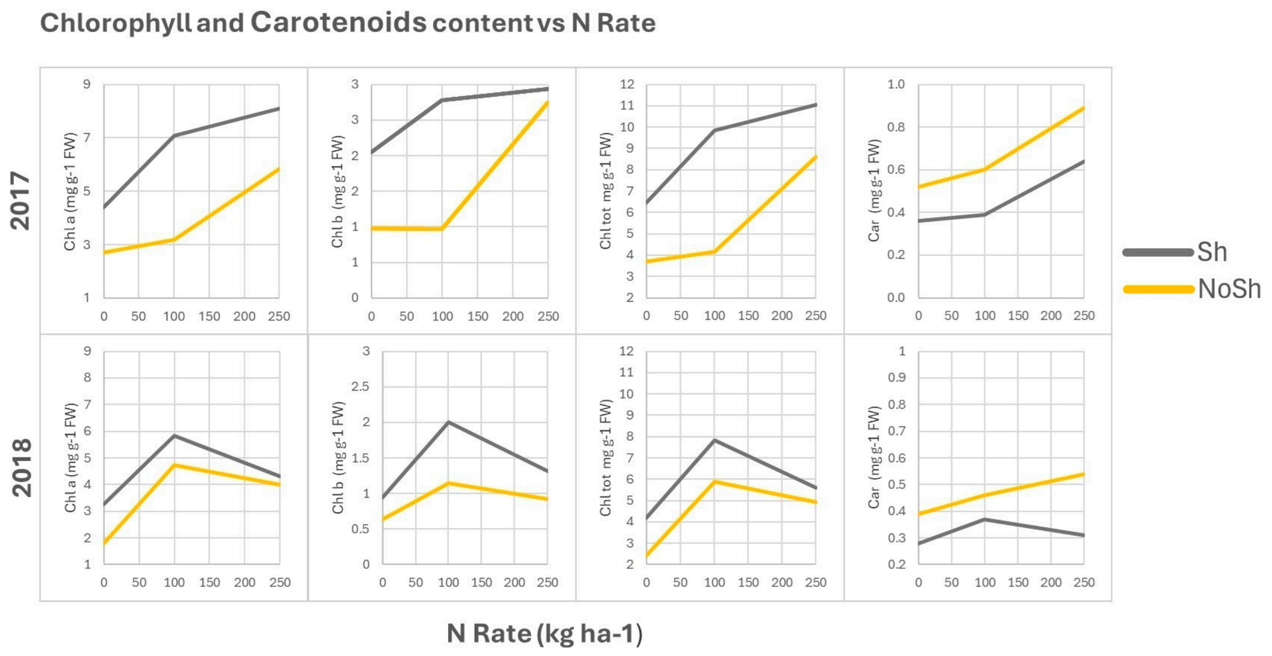

Figure S1
Interactive effects of photosynthetically active radiation (PAR) level and nitrogen application rate (N, kg ha^{-1}) on chlorophyll (Chl a, Chl b and total chlorophyll, mg g^{-1} FW) and carotenoid (Car, mg g^{-1} FW) content during two growing seasons (2017 and 2018). Yellow lines represent NoSh (no shading), and grey lines represent Sh (shading).

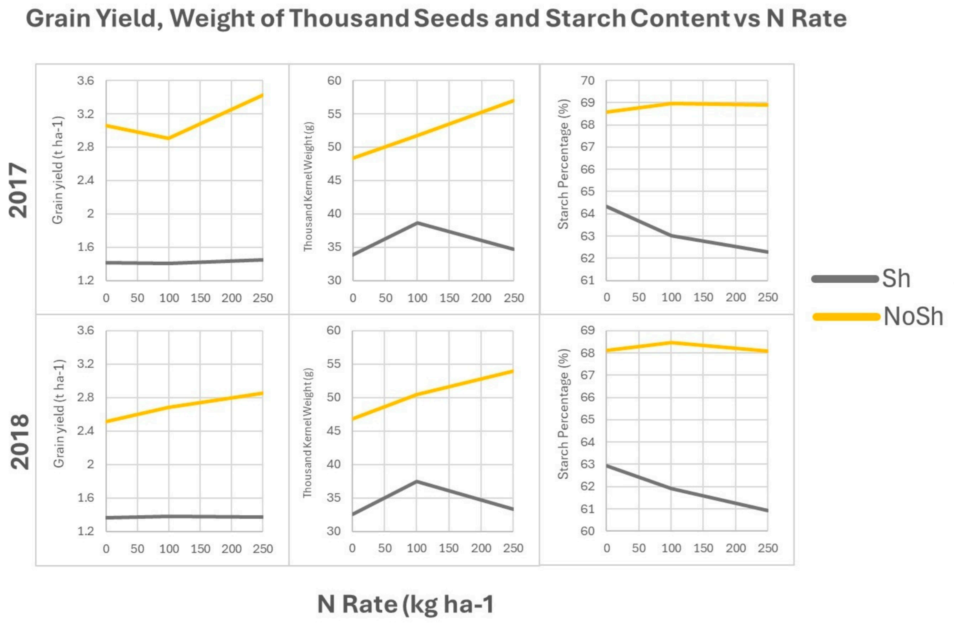

Figure S2
Interactive effects of photosynthetically active radiation (PAR) availability and nitrogen application rate (N, kg ha^{-1}) on grain yield (t ha^{-1}), thousand kernel weight (g) and starch content (%) during two growing seasons (2017 and 2018). Yellow lines represent NoSh (no shading), and grey lines for Sh (shading).

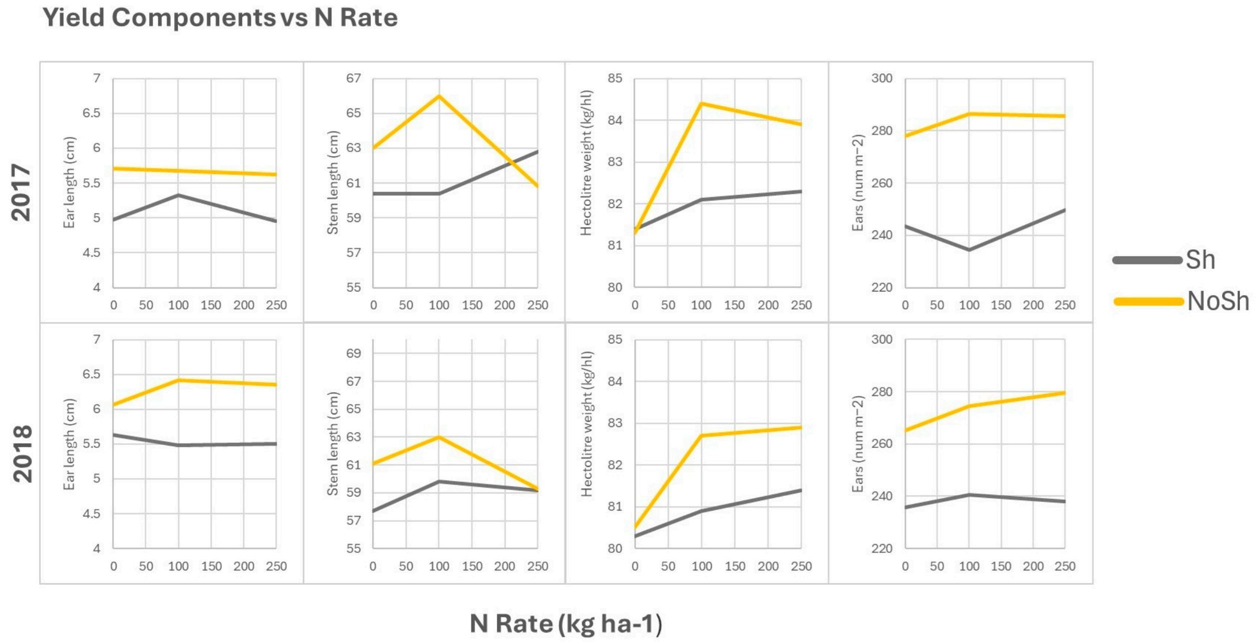

Figure S3

Interactive effects of photosynthetically active radiation (PAR) availability and nitrogen application rate (N, kg ha^{-1}) on yield components in two growing seasons (2017 and 2018), showing ear length (cm), stem length (cm), hectolitre weight (kg/hl) and ear density (num m^{-2}). Yellow lines represent NoSh (no shading), and grey lines for Sh (shading).

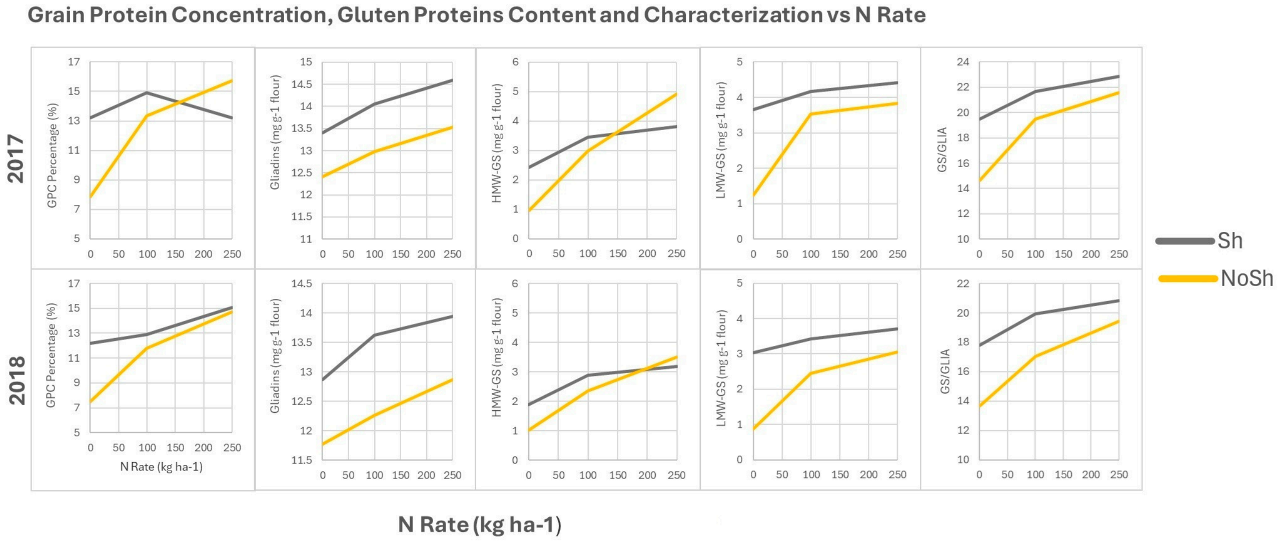

Figure S4

Interactive effects of photosynthetically active radiation (PAR) availability and nitrogen application rate (N, kg ha^{-1}) on grain protein concentration (GPC, %), gluten protein content (gliadins, HMW-GS, LMW-GS, mg g^{-1} flour) and GS/GLIA ratio in two growing seasons (2017 and 2018). Yellow lines represent NoSh (no shading), and grey lines represent Sh (shading).
